# Expression Profile Analysis to Identify Circular RNA Expression Signatures in the Prolificacy Trait of Yunshang Black Goat Pituitary in the Estrus Cycle

**DOI:** 10.3389/fgene.2021.801357

**Published:** 2022-01-24

**Authors:** Yufang Liu, Peng Wang, Zuyang Zhou, Xiaoyun He, Lin Tao, Yanting Jiang, Rong Lan, Qionghua Hong, Mingxing Chu

**Affiliations:** ^1^ Key Laboratory of Animal Genetics, Breeding and Reproduction of Ministry of Agriculture and Rural Affairs, Institute of Animal Science, Chinese Academy of Agricultural Sciences, Beijing, China; ^2^ College of Life Sciences and Food Engineering, Hebei University of Engineering, Handan, China; ^3^ Yunnan Animal Science and Veterinary Institute, Kunming, China

**Keywords:** circular RNA (circRNA) profile, prolificacy, estrus phase, pituitary gland, goat

## Abstract

The pituitary gland is an important organ. It is a complex area of the brain involved in endocrine function and reproductive regulation. However, the function of the pituitary in goat reproduction is still unclear. Herein, RNA sequencing was used to explore the expression patterns of circle RNAs (circRNAs) in the pituitary of Yunshang black goats during the various estrus phases. Then the host genes of the circRNAs were predicted, and a competing endogenous RNA (ceRNA) network was constructed. The results showed a total of 6,705 circRNAs in the pituitary of Yunshang black goats, among which 388 differentially expressed (DE) circRNAs (214 were upregulated, while 174 were downregulated) were identified between high- and low-yield Yunshang black goats in the follicular phase (HF vs. LF); moreover, 361 DE circRNAs (136 were upregulated, while 225 were downregulated) were identified between high- and low-yield Yunshang black goats in the luteal phase (HL vs. LL). There were 65 DE circRNAs targeting 40 miRNAs in the HF vs. LF comparison and 46 DE circRNAs targeting 31 miRNAs in the HL vs. LL comparison. We identified chi_circ_0030920, chi_circ_0043017, chi_circ_0008353, chi_circ_0041580, and chi_circ_0016478 as the key circRNAs through functional enrichment analysis. The ceRNA network analysis showed that chi_circ_0031209 and chi_circ_0019448 might play an important role in reproduction by influencing the expression of prolactin receptor (PRLR) in high- and low-yielding goats during the luteal phase, whereas chi_circ_0014542 regulates the expression of WNT5A during the follicular phase. Our study provided the overall expression profiles of circRNAs in the goat pituitary during the estrus phase, which provides new insight into the mechanism of high-yield goats, which can be helpful to guide goat breeding.

## Introduction

Prolificacy traits are very complex quantitative traits in goats, and they are controlled by polygenes and at multiple points (Wang et al., 2019). Previous studies showed that reproductive-related crucial genes, including *GDF9* and *BMP15*, are broadly expressed in the hypothalamus, pituitary gland, ovary, uterus, and other reproductive organs of goats (Silva et al., 2005; Pramod et al., 2013). The pituitary is the hub that connects the hypothalamus to the ovaries via the production of neuropeptides acting on pituitary hormone-producing cells. The anterior pituitary lobe releases adrenocorticotropic hormone, which stimulates the production of adrenal cortisol; thyroid-stimulating hormone, which stimulates the thyroid to produce hormones; and follicle-stimulating hormone (FSH), which works with luteinizing hormone (LH) to ensure the normal function of the ovaries (Bornstein et al., 2008). In both females and males, the FSH and LH are the same, crucial for the sexual development, maturation, and reproduction (McIlwraith and Belsham, 2020). In mammals, the pituitary, a complex area of the brain, sits in a protective bony enclosure called the sella turcica, from which it participates in the endocrine function, and reproduction regulation (Xu et al., 2020). During the estrus cycle, the hormone secretion levels from the pituitary are different during the follicular and luteal phases. The results showed that the levels of hormones, such as progesterone, estrogen, LH, and FSH, were higher during the estrus phase of the Kazakh sheep (Zhang et al., 2013). However, the regulatory mechanisms underlying reproduction in the pituitary in high- and low-yield goats are still unknown.

Circular RNA (circRNA), a common class of noncoding RNAs produced by back splicing, is an unconventional splicing event and is characterized as having no 5′-end cap structures or 3′-end polyadenylation tails (Liu et al., 2019). CircRNAs have been reported to have an endogenous role as microRNA (miRNA) sponges (Hansen et al., 2013a; Memczak et al., 2013). In previous studies, circRNAs were shown to play a significant role in the progression of a number of diseases, including Alzheimer’s disease, cardiovascular disease, diabetes, and cancer (Chen et al., 2019; Zhu et al., 2019). In mammals, the majority of tissues are regulated by circRNAs, including skeletal muscles, adipogenesis, the human frontal cortex, the thyroid gland, and the liver, and an overall enrichment of circRNA expression in the nervous system has been found (Rybak-Wolf et al., 2015). A genome-wide analysis study of circular RNAs was performed in sheep pituitary glands with prenatal and postnatal showing that many circRNAs were found by RNA-seq. Subsequently, RT-qPCR analysis demonstrated that sheep circRNAs are expressed in prenatal and postnatal pituitary glands (Li et al., 2017). A recent study used RNA-seq to identify 9,231 DE circRNAs between the estrus and anestrus pituitary systems of sheep. Then the miRNA–circRNA interaction network for these DE circRNAs that regulate sheep estrus was predicted (Li et al., 2019). There were many differences in the circRNAs expression pattern of the sheep pituitary glands during estrus and anestrus, indicating that circRNAs might be widely related to the regulation of these two states. In a study of goat cashmere fineness, four circRNAs seemed to regulate fineness when comparing Liaoning cashmere goats and Mongolia cashmere goats. However, little is known about the molecular mechanisms of the prolificacy trait in the goat pituitary gland.

Compared with other livestock, goats have many excellent production performances, and their products, including meat, milk and wool, are ubiquitous and popular worldwide. However, the global goat supply cannot satisfy the high demand in terms of quantity and quality at present (Lu and Miller, 2019). In this study, the Yunshang black goat, a domestic prolific goat breed in China, was used for circRNA profile analysis. We collected pituitary tissues from 20 high- and low-yield goats at the follicular and luteal phases. Hundreds of circRNAs were screened in the HF vs. LF and HL vs. LL comparisons. In order to further understand the relationship between circRNAs and reproduction and their important role, we also generated a regulatory network that took into account interactions between these circRNAs and miRNAs, and a competing endogenous RNA (ceRNA) network was constructed. These results might provide a new insight for prolific goat circRNAs and their potential relationship in the regulation of reproduction.

## Materials and methods

### ANIMALS AND SAMPLE PREPARATION

A native domestic goat breed, known as the Yunshang black goat, was used in this study. Twenty female goats with no significant differences in age, weight, and height were selected and grouped into either the high-yielding group (*n* = 10, average kidding number 3.00 ± 0.38, HF, and LF, five individuals per group) or the low-yielding group (*n* = 10, kidding number 1.32 ± 0.19, HL, and LL, five individuals per group) according to their litter size records (*p *< 0.05). Additionally, all goats were fed under the same conditions, with free access to water, on a goat farm in Yunnan Province. Pituitary tissues were collected from the goats at various stages during the estrus cycle, frozen immediately in liquid nitrogen, and stored at −80°C until RNA extraction.

### TOTAL RNA EXTRACTION

Total RNA for RNA sequencing (RNA-seq) was isolated from 20 pituitary samples with TRIzol reagent (Invitrogen, Carlsbad, CA, USA) according to the protocol of the manufacturer. The purity and concentration of the RNA samples were evaluated on a NanoDrop 2000 spectrophotometer (Thermo Scientific, Wilmington, DE, USA), and standard denaturing agarose gel electrophoresis was used to monitor the RNA for degradation and contamination. An RNA Nano 6000 Assay Kit with the Agilent Bioanalyzer 2100 system (Agilent Technologies, Palo Alto, CA, USA) was used to assesses the integrity of RNA. The RNA integrity number (RIN) of the samples ranged from 8.0 to 9.2, and the RIN of the samples greater than 8.0 was considered acceptable for RNA-seq.

### IDENTIFICATION AND DIFFERENTIAL EXPRESSION ANALYSIS OF CIRCULAR RNA

Twenty cDNA libraries of pituitaries from high- and low-yield goats in the follicular and luteal phases were constructed and sequenced using an Illumina HiSeq X Ten sequencer. The individuals were divided into four groups, and five replicates of each sample were considered: the high-yielding group in the follicular phase (HF-1, HF-2, HF-3, HF-4, and HF-5), the high-yielding group in the luteal phase (HL-1, HL-2, HL-3, HL-4, and HL-5), the low-yielding group in the follicular phase (LF-1, LF-2, LF-3, LF-4, and LF-5), and the low-yielding group in the luteal phase (LL-1, LL-2, LL-3, LL-4, and LL-5). FIND_CIRc and CIRI2 were used to identify the circRNAs according to previous studies (Memczak et al., 2013; Gao et al., 2017). CIRI2 searches for PCC (paired-end mapping) signals and PEM (paired-end mapping) signals and junction reads of the shear site of paired chiastic clipping according to the comparisons of the results of BWA (Li and Durbin, 2009). Then according to the results of dynamic programming alignment, circRNA read support numbers, and genome annotation information were used to filter the candidate circRNAs. Quantification was performed based on the reads that were mapped across the circularized junctions and normalized using SRPBM [number of circular reads/number of mapped reads (units in billion)/read length] (Zheng et al., 2016). Differential circRNA expression was analyzed using DESeq2 (Love et al., 2014). circRNAs with *padj* values (calibrated *p*-values) of <0.05 were considered to be significantly differentially expressed.

### QUANTITATIVE REAL-TIME POLYMERASE CHAIN REACTION VALIDATION

In order to verify the reliability of the sequencing data, eight DE circRNAs were randomly selected and confirmed by RT-qPCR with RPL19 used as the reference gene. Accounting for the characteristics of circRNA, the RT-qPCR primers were designed and are shown in Table 1. Three biological replicates of each group were analyzed using a Roche Light Cycler^®^480 II system (Roche Applied Science, Mannheim, Germany), and three duplicates of each replicate were analyzed to establish a standard curve. The following steps were applied for RT-qPCR: initial denaturation at 95°C for 5 min, followed by 40 cycles of denaturation at 95°C for 5°s and annealing at 60°C for 30°s. The data were analyzed using the 2^*−∆∆Ct*^ method.

**TABLE 1:** Information on real-time quantitative polymerase chain reaction primers and amplification product sizes of the selected circular RNAs and housekeeping gene.

Gene Name	Primer sequence	Product Length (bp)
chi_circ_0001446	F: 5’ GCATGTTAAATCGTGCTCAGG 3’	276
	R: 5’ AAATGGGTTTGGGTTCACTGA 3’	
chi_circ_0002535	F: 5’ TGGGATTCCATCGAAATCAG 3’	124
	R: 5’ ATACACAGCCTTCCGACACG 3’	
chi_circ_0006054	F: 5’ GGCTGTTACTCCCTTTCCGT 3’	168
	R: 5’ GCTCATGCGTGACCTGTCTT 3’	
chi_circ_0009732	F: 5’ GTTGACCGCCTCTTGGAAAT 3’	142
	R: 5’ ACTGGTTGGTGAGGGTTTCC 3’	
chi_circ_0018293	F: 5’ CCAGGCTTGAGAAAGGCTTAA 3’	256
	R: 5’ CAGTTATCCACCATGGCACTG 3’	
chi_circ_0022480	F: 5’ AGGAGGATGGAGGCTATGACA 3’	169
	R: 5’ TGGCACCTGAATTAACTTGCA 3’	
chi_circ_0023864	F: 5’ AGAAGCACAGAAGATGGCACA 3’	159
	R: 5’ ATGTCATACCGAGAGGCACGT 3’	
chi_circ_0029559	F: 5’ ATACAGCAAATGGAAGCCCA 3’	238
	R: 5’ TTCAGGCACTCCTCTCTGGC 3’	
chi_circ_0037447	F: 5’ TTCCCAAACTGGCTTGTCTG 3’	186
	R: 5’ GATACCTCGGTTGCTCCTGC 3’	
chi_circ_0037872	F: 5’ ATTCCTAAGACGGACTGCAGG 3’	88
	R: 5’ TTCATCCGACTCCTCAGAAGC 3’	

### INTEGRATED FUNCTIONAL ENRICHMENT ANALYSIS

Functional annotation of host genes with DE circRNA was based on the GO and KEGG annotation of the source genes. GO annotation was performed based on the corresponding genes in NCBI and their GO annotations. This information was held in the following database: https://ftp.ncbi.nlm.nih.gov/gene/DATA/gene2go.gz. For KEGG annotation, we used the KOBAS software to test the statistical enrichment of host genes with DE circRNAs in the KEGG pathways (Xie et al., 2011). A threshold of *p* < 0.05 was used as a criterion for the determination of whether the enrichment analysis was significant.

### CONSTRUCTION OF PREDICTED COMPETING ENDOGENOUS RNA NETWORKS AND ENRICHMENT ANALYSIS

According to the data of the differentially expressed mRNA, miRNA, and circRNA transcripts, miRanda (v3.3a) (Marks et al., 2005) was used to identify biological targets of each miRNA from the protein-coding and circRNA transcripts that showed a significantly negative correlation with miRNA expression. Then the protein-coding transcript miRNA and circRNA transcript miRNA pairs were obtained, and the competing endogenous RNA (ceRNA) network was constructed. The Pearson correlation coefficient of the miRNAs, the target mRNAs, and the target circRNAs <−0.8 were selected for analysis.

### STATISTICAL ANALYSIS

All data were analyzed by SPSS 17.0 statistics software, and the mean of three replicates was evaluated and is displayed as the mean ± standard error (SE). Significance was determined using *t-*tests and is presented as ∗*p* < 0.05 and ∗∗*p* < 0.01.

## Results

### SUMMARY OF CIRCRNAS FROM THE GOAT PITUITARY

**FIGURE 1:**
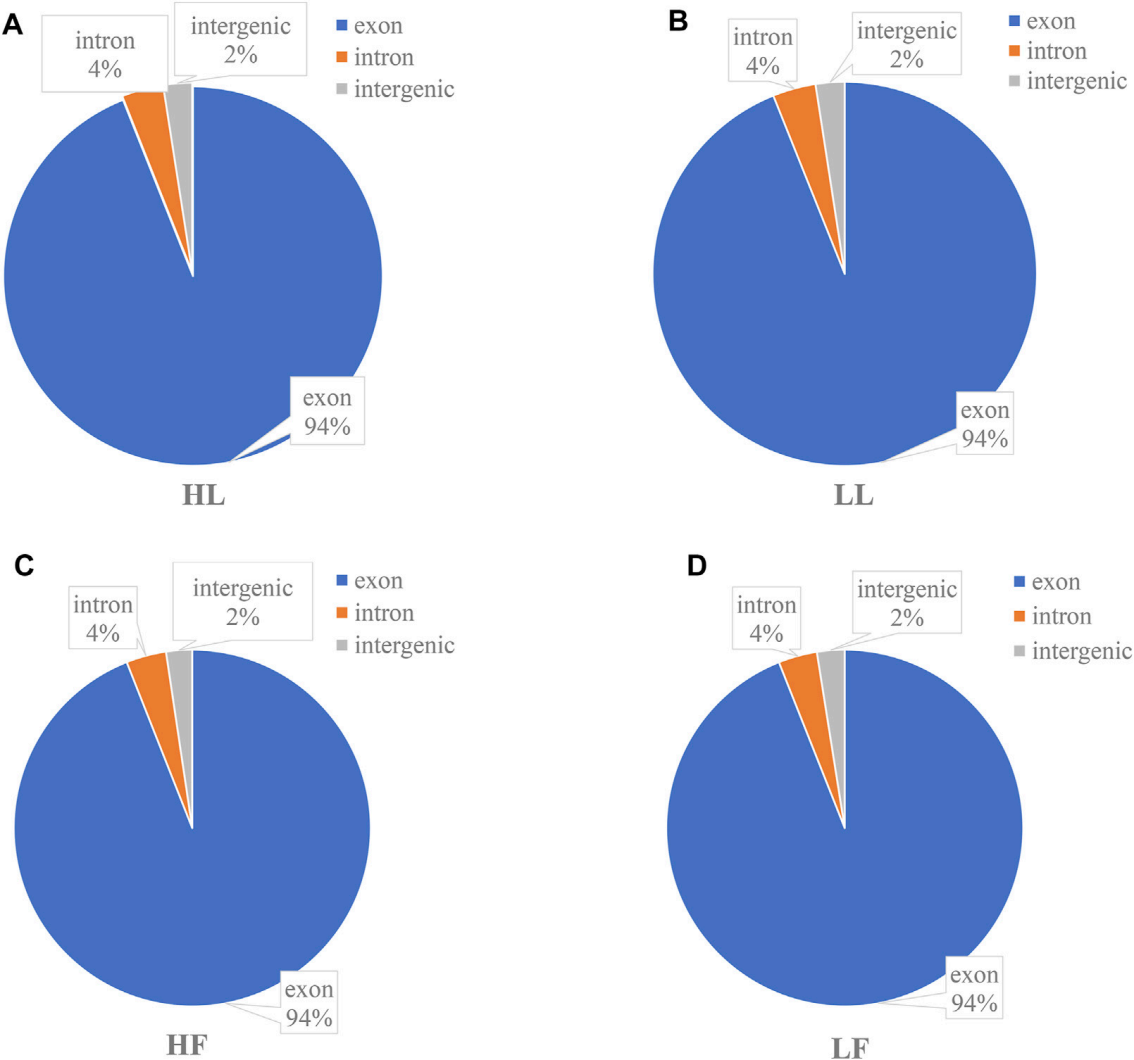
The functional and regional distribution of identified circular RNAs. **(A)** The functional region distribution of circular RNAs identified in high-yield goats in the luteal phase, HL; **(B)** low-yield goats in the luteal phase, LL; **(C)** high-yield goats in the follicular phase, HF; and **(D)** low-yield goats in the follicular phase, LF.

**FIGURE 2:**
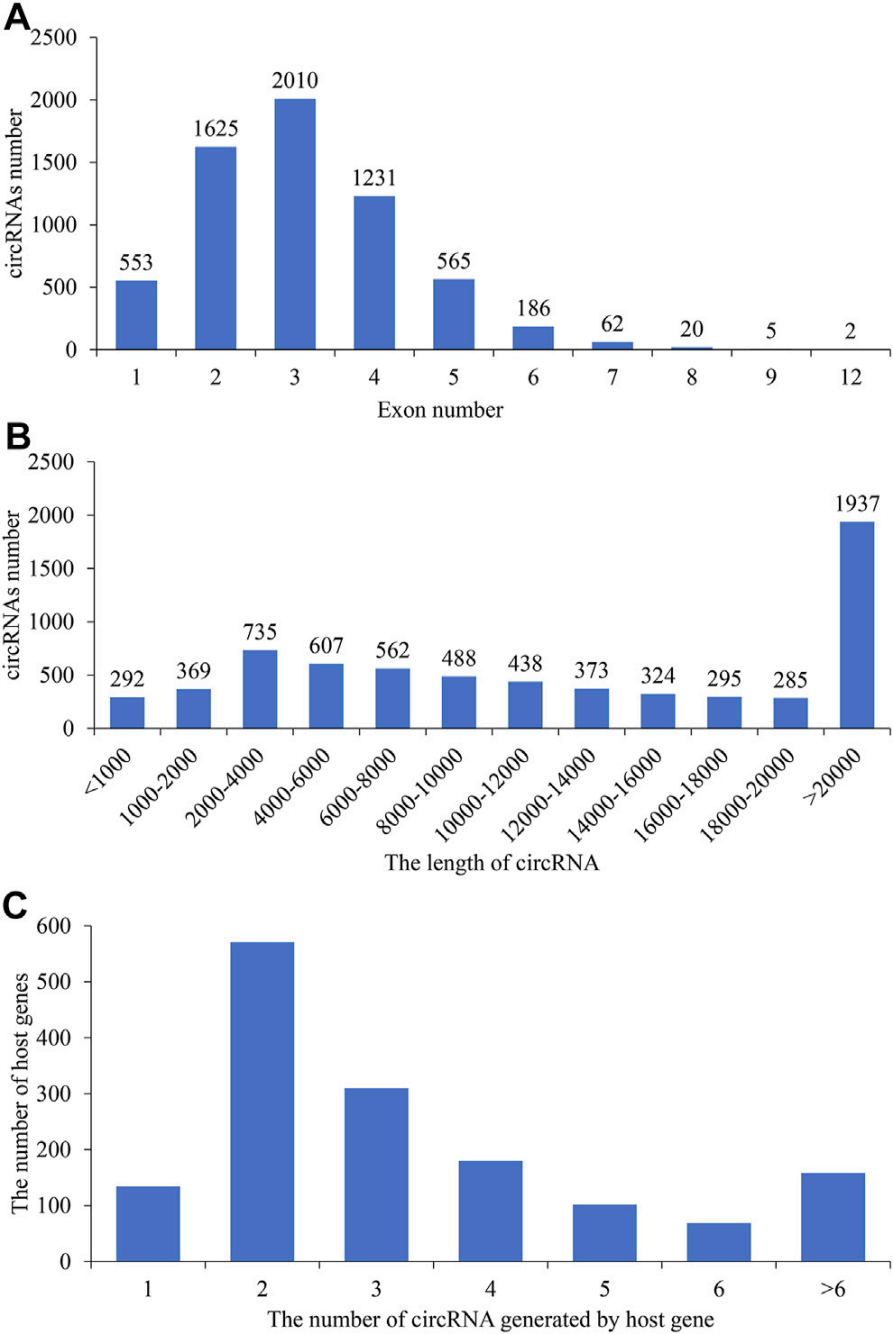
Statistics of the exon number and length of identified circular RNAs (circRNAs). The exon number **(A)** and length **(B)** distributions of circRNAs; **(C)** the number of circRNAs generated by host genes.

To understand the differentially expressed circRNAs in the goat pituitary, a total of 359.8 Gb raw data were obtained after sequencing, and after deleting the low-quality raw reads, the mapping ratios of clean reads were 74.03%, 76.32%, 81.17%, and 76.42% in the HL, LL, HF, and LF groups, respectively (Supplementary Table S1). A total of 6,705 circRNAs were identified in 20 pituitary tissues (Supplementary Table S2), a lot of which were distributed on chromosome 1, and then followed by chromosomes 3 and 10 (Supplementary Figure S1). We found that the genomic loci from which the circRNAs were derived were located in 29 autosomes and X chromosomes in the four groups. CircRNAs were also identified in diverse functional regions, including exons, introns, and intergens, and the majority of which were identified in exon regions (Figure 1). Moreover, the four goat groups, namely, HL (Figure 1A **)**, LL (Figure 1B), HF (Figure 1C), and LF (Figure 1D), exhibited similar circRNA distributions and percentages. The lengths of circRNAs ranged from 1,000 to 20,000 bp among the four groups (Figure 2B), and the majority of circRNAs contained two to four exons (Figure 2A). In this study, a total of 6,705 circRNAs screened were derived from 2,730 host genes. Almost half of these host genes (49%) generated only one circRNA, and 21% of these host genes generated two circRNAs, whereas 5.8% of the host genes generated more than six circRNAs (Figure 2C).

### ANALYSIS OF DIFFERENTIALLY EXPRESSED CIRCULAR RNA EXPRESSION PROFILES IN THE GOAT PITUITARY

**FIGURE 3:**
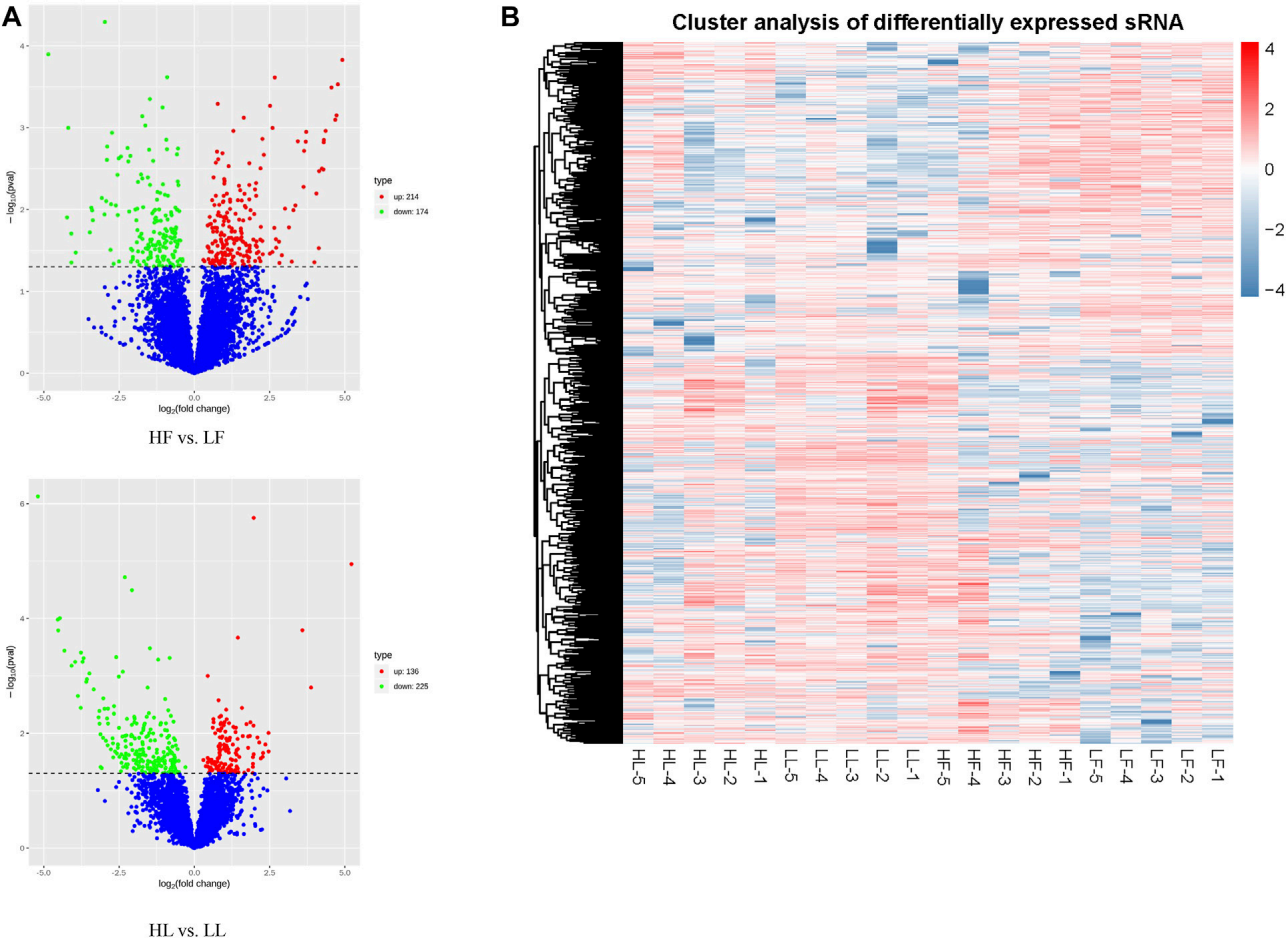
Overview of differentially expressed circular RNAs (DE circRNAs). **(A)** A volcanic plot of high-yielding goats versus low-yielding goats in the follicular phase (HF vs. LF) and the luteal phase (HL vs. LL). **(B)** The expression pattern of DE circRNAs and hierarchical clustering analysis in the HF, LF, HL, and LL groups.

**FIGURE 4:**
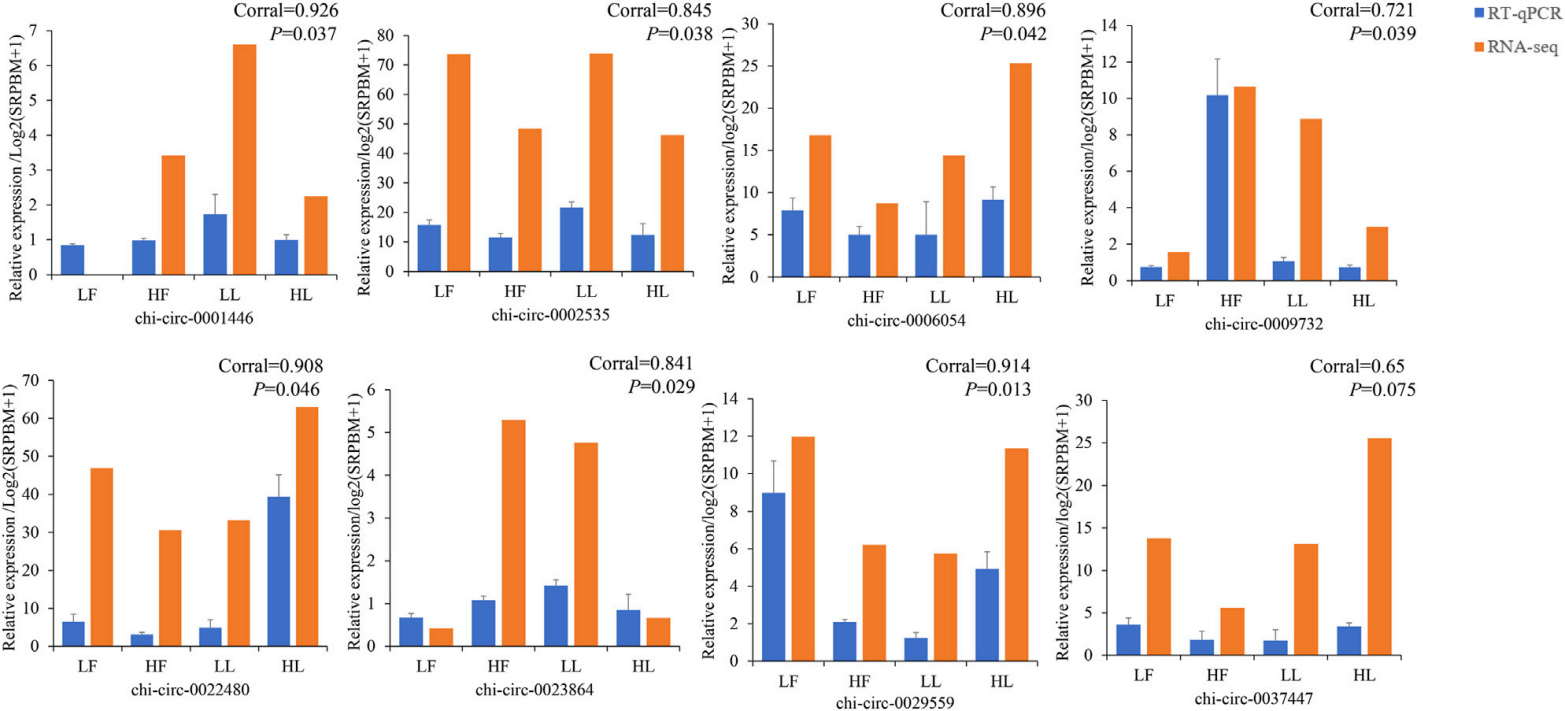
Validation results of eight selected circular RNAs in the four groups (HF, LF, HL, and LL) by real-time quantitative polymerase chain reaction (RT–qPCR). Corral represents the correlation coefficient.

According to the normalized expression with |log2 (fold change)| > 1 and *p-value* < 0.05, 388 DE circRNAs (214 upregulated and 174 downregulated) and 361 DE circRNAs (136 upregulated and 225 downregulated) were identified in the HF vs. LF and HL vs. LL comparisons, respectively (Figure 3A; Supplementary Table S3). Then, we used a cluster heatmap analysis of differentially expressed circRNAs to better understand their potential relationship (Figure 3B). To ensure the accuracy of the RNA-seq strategy, eight differentially expressed circRNAs were randomly selected, and specific RT-qPCR primers were designed within the circRNA junction regions as previously described (Zheng et al., 2020). The expression levels of circRNAs determined by RT-qPCR and RNA-seq were highly consistent (Figure 4). This means there is significant reliability of the RNA-seq data acquisition and subsequent analysis procedures in this study. The results showed that the host genes of chi_circ_0036753, chi_circ_0012160, and chi_circ_0040916 were *TGFBR2*, *ANK2*, and *MAPK8* in the HF vs. LF comparison; the host genes of chi_circ_0038881 and chi_circ_0031910 were *ADCY9*, *GNAO1*, and *PLCB1* in the HL vs. LL comparison.

### ANALYSIS OF GENE ONTOLOGY AND KYOTO ENCYCLOPEDIA OF GENES AND GENOMES PATHWAYS

**FIGURE 5:**
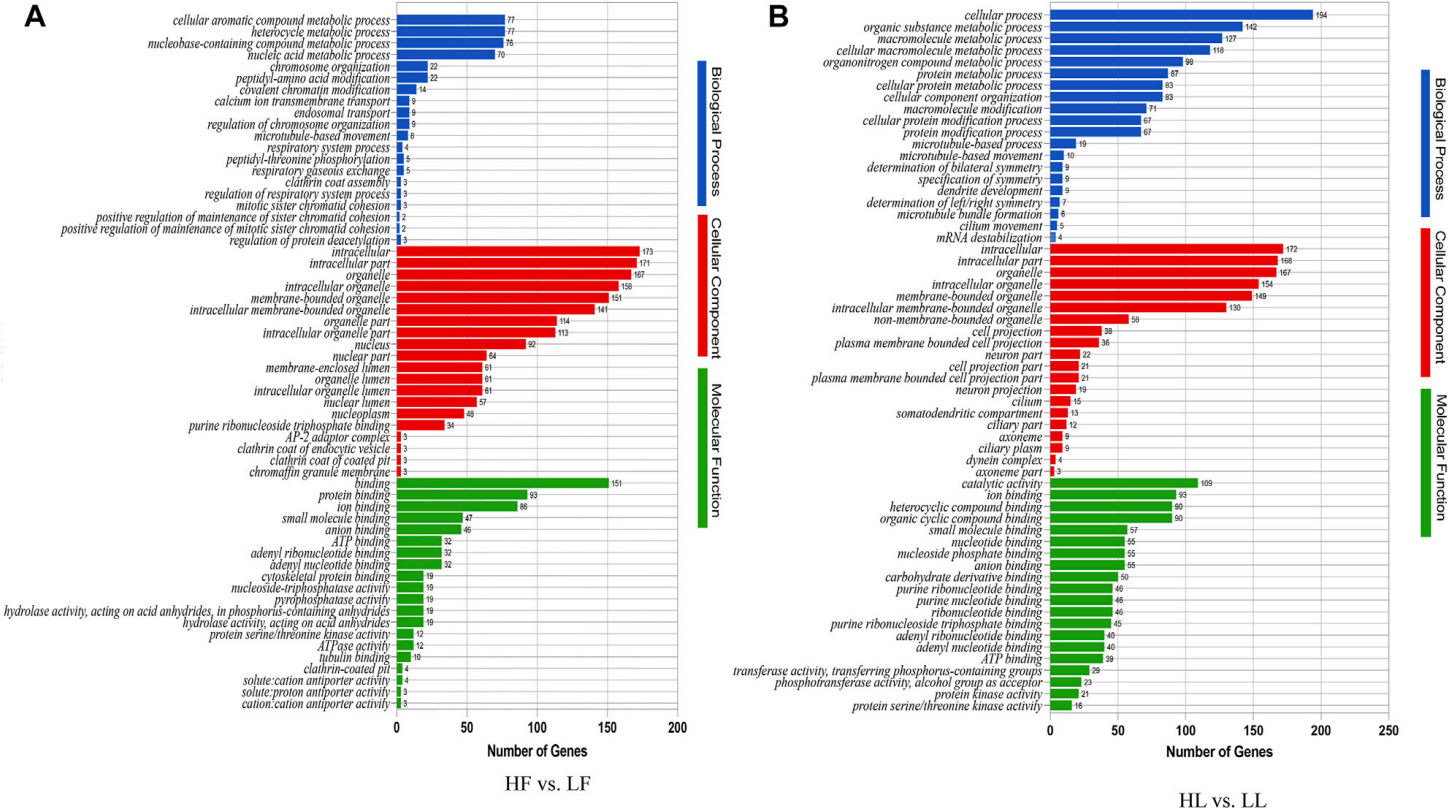
Gene ontology functional enrichment analysis. **(A)** The enriched GO terms in biological process, molecular function, and cellular component in high-yielding goats in the follicular phase versus low-yielding goats in the follicular phase (HF vs. LF). **(B)** The GO terms enriched in biological process, molecular function, and cellular component in high-yielding goats in the luteal phase versus low-yielding goats in the luteal phase (HL vs. LL). Note. *p *< 0.05.

**FIGURE 6:**
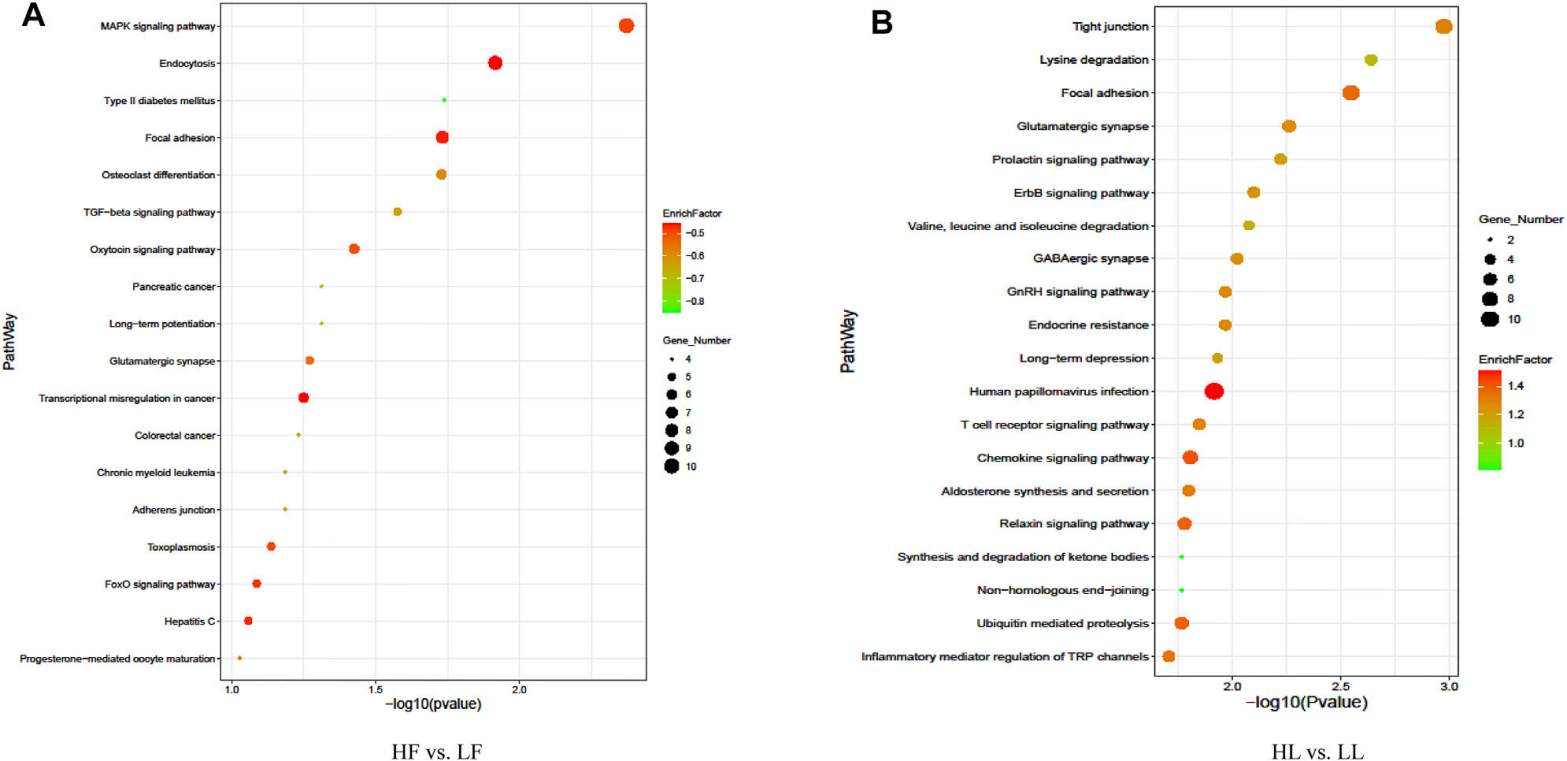
Kyoto Encyclopedia of Genes and Genomes pathway enrichment analysis. **(A)** The pathways enriched in high-yielding goats in the follicular phase versus low-yielding goats in the follicular phase (HF vs. LF). **(B)** The pathways enriched in high-yielding goats in the luteal phase versus low-yielding goats in the luteal phase (HL vs. LL). Note. *p *< 0.05.

In order to better study the functions of the DE circRNAs, the GO term and KEGG pathway were analyzed. The analysis of GO enrichment showed that the top enriched GO terms between HF and LF were cellular processes in the main category of biological processes, binding in molecular function, and cell in the cellular component (Figure 5A; Supplementary Table S4). Meanwhile, the top enriched GO terms in HL vs. LL were similar to those in the HF vs. LF comparison and the number of enriched genes (Figure 5B; Supplementary Table S4). The analysis of KEGG enrichment showed that the most enriched pathway in HF vs. LF was the MAPK signaling pathway, and the progesterone-mediated oocyte maturation related to reproduction was also enriched (Figure 6A, Supplementary Table S5). About the HL vs. LL comparison, the most abundant pathway was focal adhesion. Several pathways related to reproduction, including the GnRH signaling pathway and the estrogen signaling pathway, were also enriched (Figure 6B, Supplementary Table S5).

### Analysis of the targeting relationship between circular RNAs and microRNAs

**FIGURE 7:**
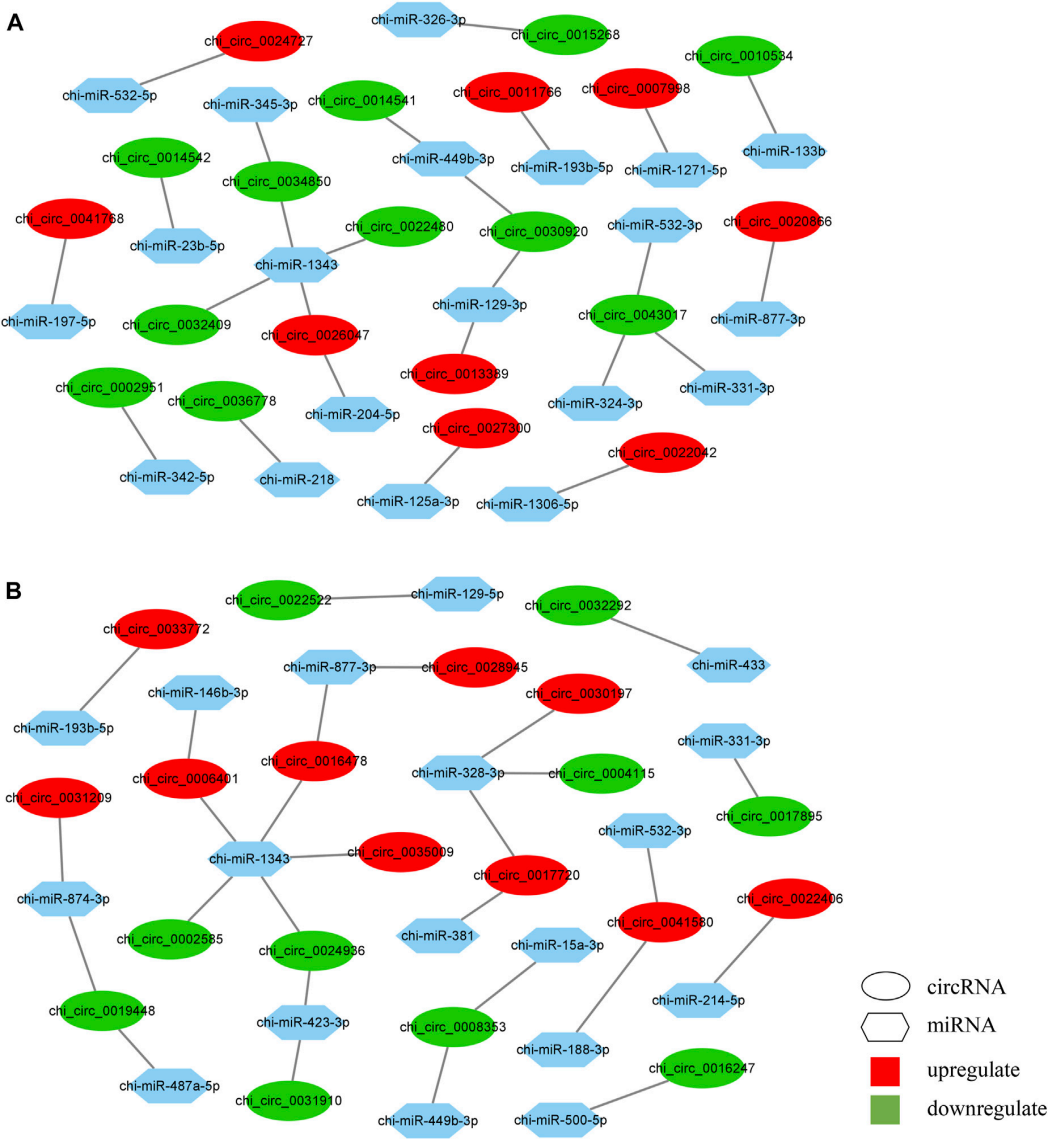
Prediction of microRNA-circular RNA interactive networks. **(A)** The differentially expressed circular RNAs (DE circRNAs) in high-yielding goats in the follicular phase versus low-yielding goats in the follicular phase (HF vs. LF). **(B)** The differentially expressed circular RNAs (DE circRNAs) in high-yielding goats in the luteal phase versus low-yielding goats in the luteal phase (HL vs. LL).

To further identify the key circRNA in the prolificacy trait of goats, the binding sites of miRNA-bound circRNAs were predicted by miRanda software. We identified 40 miRNAs that bind to 65 DE circRNAs from the HF vs. LF comparison, and 31 miRNAs that bind to 46 DE circRNAs from the HL vs. LL comparison. Consistent with the results of a previous study, our results also showed one-to-many and many-to-one characteristics of miRNA–circRNA (Figure 7; Supplementary Table S6) (Zhang et al., 2019). Among these, miR-133a-b, miR-129-3p, and miR-324-3p were identified in the HF vs. LF comparison only, while miR-129-5p was identified in the HL vs. LL comparison. The function of these miRNAs has mainly impacted reproduction; thus, their target circRNAs were likely associated with reproduction.

### INTEGRAL COMPETING ENDOGENOUS RNA ANALYSIS

To further detect the potential function of the DE circRNAs, the circRNA–miRNA interactive networks were constructed in the two comparisons. In total, 25 circRNA–miRNA pairs were used in the HF vs. LF comparison (Figure 7A), and 27 pairs were used in the HL vs. LL comparison (Figure 7B). In our results, five circRNAs were the core of the circRNA–miRNA network, of which chi_circ_0030920 targeted by chi-miR-129-3p and chi_circ_0043017 targeted by chi-miR-324-3p were identified in the HF vs. LF comparison, chi_circ_0008353 targeted by chi-miR-15a-3p, chi_circ_0041580 targeted by chi-miR-188-3p, and chi_circ_0016478 targeted by chi-miR-1343 were identified in the HL vs. LL comparison.

**FIGURE 8:**
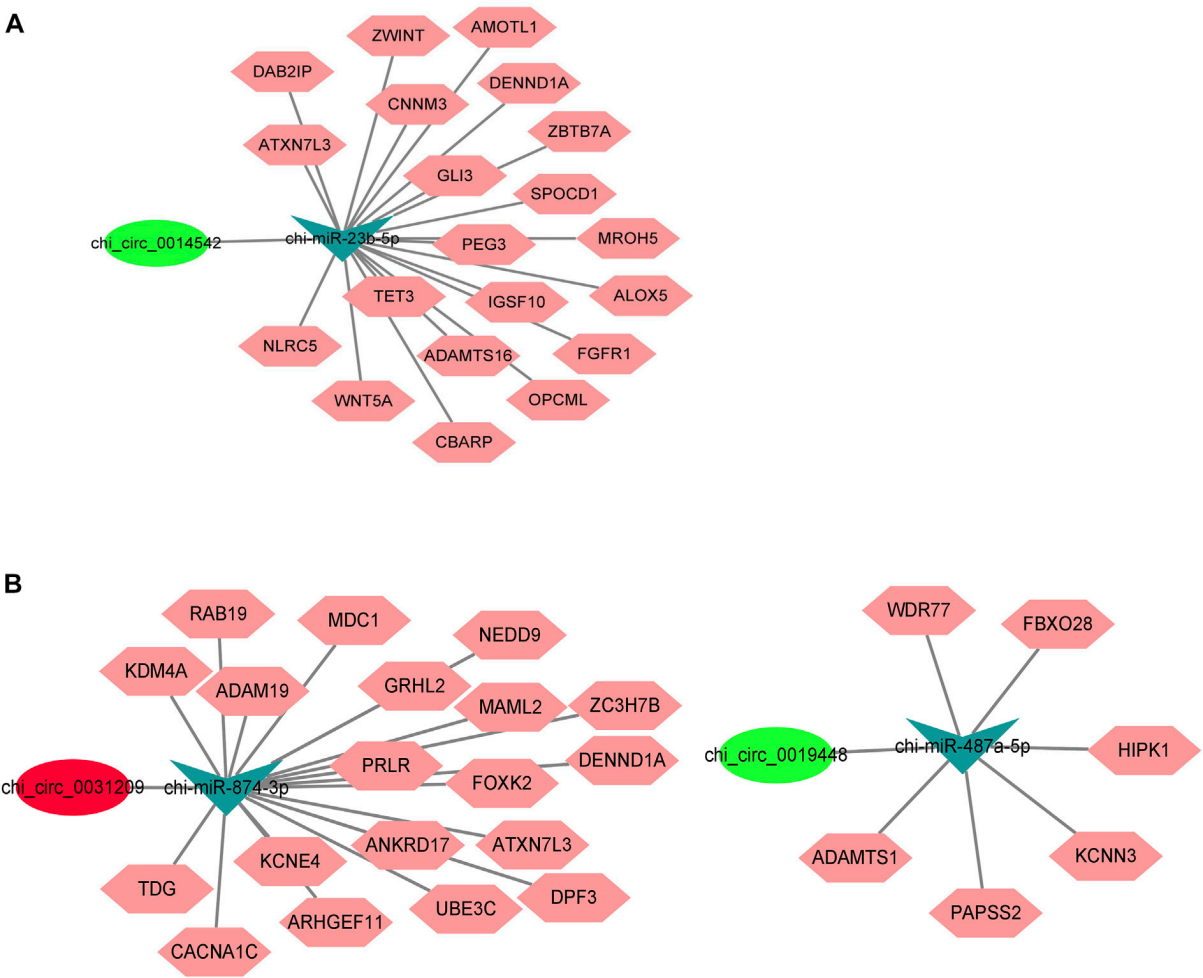
Predicted competing endogenous RNA (ceRNA) network involving **(A)** chi_circ_0014542, **(B)** chi_circ_0031209 and chi_circ_0019446.

We also constructed ceRNA networks involving chi_circ_0031209, chi_circ_0019448 (HL vs. LL comparison), and chi_circ_0014542 (HF vs. LF comparison) in the two comparisons. We searched the potential binding miRNAs and target genes of the three circRNAs from the TargetScan database and selected the target genes related to reproductive traits to construct a ceRNA network (Figure 8; Supplementary Table S7).

## Discussion

The goat is an important agricultural animal and provides meat, fleece, and milk. The kidding number per birth is a restriction for goat production. Normally, the average kidding number of goats is two. A lot of local goat breeds have low prolificacy in China. The Yunshang black goat is one of the most prolific goat breeds in China and is an important goat breed in the rural economy (Tao et al., 2021). The fecundity traits of mammals are determined by complex regulatory factors, including genetic material, nutrient levels, feeding environment, and management. In goats, the main genes affecting prolificacy traits have not been found. In a previous study, many candidate genes with SNPs were found to be associated with the kidding number of goats. For example, the SNPs of *ASMT* (*acetylserotonin O-methyltransferase*) and *ADAMTS1* (*ADAM metallopeptidase with thrombospondin type 1 motif*) are essential for improving the litter size of the Jining Gray goats (Hu et al., 2020). mRNA and miRNA integration analyses were also performed in the ovarian follicles (large follicles and small follicles) of uniparous and multiple Chuanzhong black goats during the estrus phase with RNA sequencing (RNA-seq) (Zou et al., 2020). In this study, the mRNA–miRNA interaction network analysis suggested that five pairs might be related to fertility, including *CYP11A1* (miR-122), *SERPINA5* (miR-1, miR-206, miR-133a-3p, and miR-133b), and *PTGFR* (miR-182, miR-122). These results will provide a potential function for goat prolificacy traits. In previous studies, much of the research on prolificacy traits in goats has focused on the analysis of differentially expressed circRNAs, such as the ovary and uterus (Zhang et al., 2017; Liu et al., 2018; Zou et al., 2020). However, the pituitary gland plays an equally important role as an important component of the gonadal axis.

The pituitary gland is a key endocrine organ that regulates the secretion of reproductive-related hormones (Weltzien et al., 2004; Zohar et al., 2010). Located in the anterior pituitary (adenohypophysis), gonadotropes produce and release the two major gonadotropins (follicle-stimulating and luteinizing hormones, FSH and LH, respectively) into the blood circulation, which stimulate gonadal gametogenesis, and steroidogenesis (Pierce and Parsons, 1981). As the central part of the brain–pituitary–gonadal (BPG) axis, pituitary gonadotropes play a crucial role in the reproductive function of all vertebrates (Santiago-Andres et al., 2021). In recent years, the function of noncoding RNAs was found to be related to reproduction in mammals, especially circRNAs (Zhang et al., 2019). In a recent study, some evidence showed that circRNAs are regulators of the fecundity of mammals (Cheng et al., 2017; Zou et al., 2020). In GnRH-treated rats, a previous study identified 14 DE circRNAs that may act on the secretion and regulation of reproductive hormones (Shen et al., 2019). In a previous study of dairy goats, differentially expressed circRNAs were found in the endometrium, which may be involved in the formation of endometrial receptivity (Shang et al., 2021). Recent advances in circRNAs in the sheep pituitary have provided new insights into the complexity of reproduction (Li et al., 2019). Considering the lack of detailed circRNA expression profiles in the goat pituitary gland, determining the expression profiles of circRNAs and their potential functions in the pituitary gland may also improve our understanding of goat reproduction. Our data showed that among 6,677 circRNAs in the goat pituitary in the four groups, 94% were located in the exon region, which was higher than that in the goat skin (Zheng et al., 2020). Therefore, our results also confirmed the conclusion that circRNAs might be specific to particular tissues or breeds (Zhang et al., 2019).

The function of circRNAs identified thus far is to competitively bind miRNAs to act as sponges and thereby regulate the expression of their downstream target genes (Tang and Hann, 2020). In our study, there were 388 and 361 DE circRNAs between high- and low-yield goats during the follicular phase and luteal phase, respectively. Nineteen circRNAs commonly expressed between the two comparisons were identified, of which eight circRNAs were selected for detection by RT-qPCR. This result suggested that the more highly regulated processes during kidding occur in the follicular phase. GO analysis of the two comparisons indicated that most DE circRNAs were involved in intracellular, binding, and cellular aromatic compound metabolic processes in HF vs. LF, and cellular processes and intracellular and catalytic activities in HL vs. LL. Meanwhile, the analysis of KEGG pathway also indicated that circRNAs participate in signaling pathways directly related to pituitary gland functions, including the TGF-beta signaling pathway (Hata and Chen, 2016), progesterone-mediated oocyte maturation (Cao et al., 2019), the MAPK signaling pathway (Gao et al., 2021), and the FoxO signaling pathway (Huang et al., 2016), in the HF vs. LF comparison. In the HL vs. LL comparison, the DE circRNAs were primarily involved in the GnRH signaling pathway (Flanagan and Manilall, 2017), estrogen signaling pathway (Fuentes and Silveyra, 2019), and prolactin signaling pathway (Augustine et al., 2017). In particular, *MAPK8*, *MAPK1*, *BRAF*, and *MAD1L1* were enriched during progesterone-mediated oocyte maturation in the goat pituitary in the HF vs. LF comparison, *MAPK8*, *ADCY9*, *SRC*, *PLCB1*, and *SOS2* were enriched in the GnRH signaling pathway, and *GNAO1*, *ADCY9*, *SRC*, *PLCB1*, and *SOS2* were enriched in the estrogen signaling pathway in the HL vs. LL comparison. These genes are important for hormone production and mediate oocyte maturation, and these circRNAs are probably related to the goat prolificacy trait. All of these predicted target genes indicate that the regulation of the estrus in goats is a process involving comprehensive regulation of prolificacy biological pathways, and more attention needs to be paid to these in the future.

CeRNA network analysis is the core of circRNA profiles (Hansen et al., 2013b). Recent studies have found that a negative correlation between circRNA and miRNA was demonstrated by downregulation of miRNA and upregulation of circRNA or vice versa, which confirmed the sponge function of the circRNA (Lai et al., 2014; Li et al., 2015). Therefore, 76 and 55 circRNA–miRNA pairs were predicted in the HF vs. LF and HL vs. LL comparisons, respectively, and a large and complex network of interactions could be formed. However, the function of circRNA–miRNA in the reproductive process needs further validation. In our constructed ceRNA network, chi_circ_0014542 was shown to be a sponge for chi-miR-23b-5p, which targets 20 genes, including the reproductive-associated genes *WNT5A* and *FGFR1* (Yuan et al., 2019; Azimian-Zavareh et al., 2021). During the luteal phase, chi_circ_0031209-chi-miR-874-3p and chi_circ_0019448-chi-miR-487a-5p were identified, and they targeted 19 genes and 6 genes, respectively. Interestingly, the *PRLR* played an important role in the network. Prolactin receptor (PRLR) has both pro- and antigonadal roles in the regulation of avian ovarian functions (Hu et al., 2017). In addition, *PRLR* can also be activated by three sequence-diverse hormones: prolactin, GH, and placental lactogen (Brooks, 2012), suggesting that chi_circ_0031209-chi-miR-874-3p-*PRLR* plays a key role in the goat pituitary gland during the luteal phase. Overall, these circRNA–miRNA–mRNA pairs may regulate the function of the pituitary between high- and low-yielding conditions throughout estrus, although the specific mechanisms need further study in the future (John et al., 2005, Westholm et al., 2014).

## Conclusion

In summary, we established the circRNA expression profile of the goat pituitary gland. We also identified some key circRNAs, including chi_circ_0030920, chi_circ_0043017, chi_circ_0008353, chi_circ_0041580, and chi_circ_0016478, through functional enrichment analysis, and chi_circ_0031209, chi_circ_0019448, and chi_circ_0014542, through a ceRNA network. Our study presents an integral circRNA analysis of the goat pituitary gland and provides a reference for understanding goat prolificacy.

## Data Availability

The datasets presented in this study can be found in online repositories. The names of the repository/repositories and accession number(s) can be found in the article/Supplementary Material.
