# Supplementary figures and images for: Expression Profile Analysis to Identify Circular RNA Expression Signatures in the Prolificacy Trait of Yunshang Black Goat Pituitary in the Estrus Cycle
Source: Front Genet. 2022 Jan 24;12:801357. doi: 10.3389/fgene.2021.801357 (PMC8820483; doi:10.3389/fgene.2021.801357)

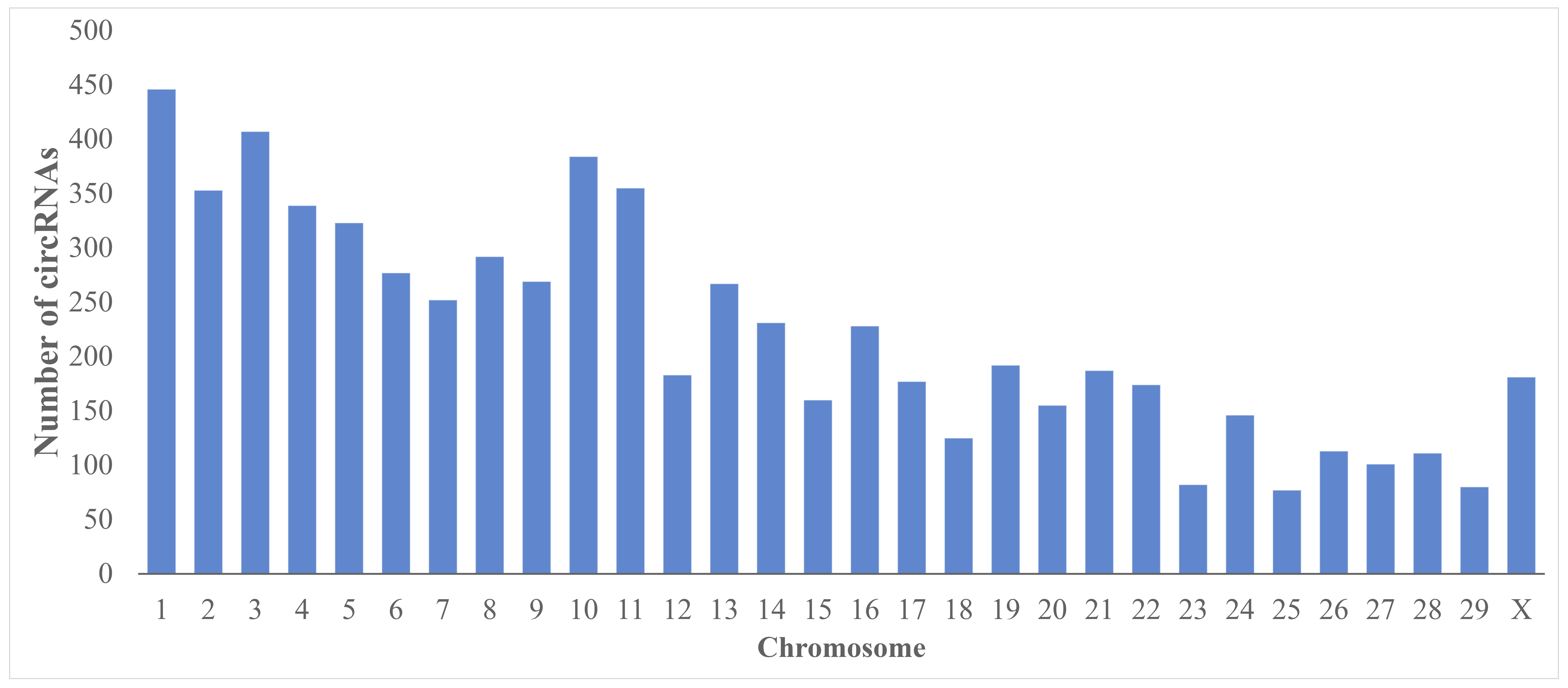

Supplement: Supplementary file 3 [file Image1.tif]
